# The Impact of Synaptic Zn^2+^ Dynamics on Cognition and Its Decline

**DOI:** 10.3390/ijms18112411

**Published:** 2017-11-14

**Authors:** Atsushi Takeda, Hanuna Tamano

**Affiliations:** Department of Neurophysiology, School of Pharmaceutical Sciences, University of Shizuoka, 52-1 Yada, Suruga-ku, Shizuoka 422-8526, Japan; tamano@u-shizuoka-ken.ac.jp

**Keywords:** Zn^2+^ signaling, hippocampus, memory, Ca^2+^ signaling, perforant pathway, dentate granule cell

## Abstract

The basal levels of extracellular Zn^2+^ are in the range of low nanomolar concentrations and less attention has been paid to Zn^2+^, compared to Ca^2+^, for synaptic activity. However, extracellular Zn^2+^ is necessary for synaptic activity. The basal levels of extracellular zinc are age-dependently increased in the rat hippocampus, implying that the basal levels of extracellular Zn^2+^ are also increased age-dependently and that extracellular Zn^2+^ dynamics are linked with age-related cognitive function and dysfunction. In the hippocampus, the influx of extracellular Zn^2+^ into postsynaptic neurons, which is often linked with Zn^2+^ release from neuron terminals, is critical for cognitive activity via long-term potentiation (LTP). In contrast, the excess influx of extracellular Zn^2+^ into postsynaptic neurons induces cognitive decline. Interestingly, the excess influx of extracellular Zn^2+^ more readily occurs in aged dentate granule cells and intracellular Zn^2+^-buffering, which is assessed with ZnAF-2DA, is weakened in the aged dentate granule cells. Characteristics (easiness) of extracellular Zn^2+^ influx seem to be linked with the weakened intracellular Zn^2+^-buffering in the aged dentate gyrus. This paper deals with the impact of synaptic Zn^2+^ signaling on cognition and its decline in comparison with synaptic Ca^2+^ signaling.

## 1. Introduction

Cognitive activity has been closely linked to strengthening and weakening synaptic connections between neurons that is synaptic plasticity such as long-term potentiation (LTP) and long-term depression (LTD). The hippocampal formation, which spans the posterior-to-anterior extent of the base of the temporal lobes, plays a key role in learning, memory, and recognition of novelty [1]. In its transverse axis, the hippocampal formation consists of the entorhinal cortex, the dentate gyrus, the CA3 and the CA1 subfields, and the subiculum. The entorhinal cortex functions as the gateway into the hippocampal formation [2]. The entorhinal cortex layer II projects to dentate granule cells via the perforant pathway, and dentate granule cells project to CA3 pyramidal cells via the mossy fibers. CA3 pyramidal cells interconnect with other CA3 neurons and project to CA1 pyramidal cells via the Shaffer collaterals. Finally, CA1 pyramidal cells connect to the subiculum. The entorhinal cortex layer II also projects to CA3 pyramidal cells, and the entorhinal cortex layer III also projects to CA1 pyramidal cells and the subiculum [3].

In the process of changes in synaptic structure for memory formation, glutamatergic neurons play a key role in the main neural circuit of the hippocampal formation. Research on synaptic plasticity opens a window for the molecular mechanisms of memory. Changes in both presynaptic and postsynaptic strength have been implicated in the mechanisms of LTP and LTD, and attention has been paid to changes in postsynaptic glutamate receptor density [4]. Intracellular Ca^2+^ signaling via the network of signaling molecules controls the glutamate receptor density and induces synaptic signals, as is observed in learning situations [5], followed by cognitive performance and memory.

For *N*-methyl-d-aspartate (NMDA)-receptor-dependent plasticity, the influx of extracellular Ca^2+^ into postsynaptic neurons through NMDA receptors plays a key role [6]. However, glutamate receptor activation by excess of extracellular glutamate, which is known as glutamate excitotoxicity [7,8], leads to a final common pathway for neuronal death and is linked with pathophysiological processes of neurological disorders [9,10]. CA1 pyramidal cells are the most vulnerable to neurodegeneration in the hippocampus after stroke/ischemia [11,12,13]. A well-known fact is that extracellular Ca^2+^ influx into postsynaptic neurons, in addition to Ca^2+^ release from the calcium stores results in neuronal death.

On the other hand, extracellular glutamate signaling also induces cellular transients in Zn^2+^ concentration, i.e., intracellular Zn^2+^ signaling, which is required for synaptic plasticity [14,15] and may have crosstalk to intracellular Ca^2+^ signaling via calcium channels [16,17]. On the basis of the subsequent evidence that glutamate-induced neuronal death is due to extracellular Zn^2+^ influx into postsynaptic neurons, which is dynamically linked with Zn^2+^ release from zincergic neurons, a subclass of glutamatergic neurons that concentrate zinc in the presynaptic vesicles [18,19,20,21,22], this paper deals with the impact of synaptic Zn^2+^ signaling on cognitive function and dysfunction in comparison with synaptic Ca^2+^ signaling. While the hydrated Ca^2+^ ion is the major species in intracellular Ca^2+^ signaling, this is not the case in intracellular Zn^2+^ signaling because the Zn^2+^ ion has much higher affinities for donors of ligands [23]. The Zn^2+^ ion is different from the Ca^2+^ and Mg^2+^ ions because it forms much stronger complexes with water and various anions and ligands. These characteristics are important for its synaptic functions.

## 2. Physiology of Brain Zn^2+^

**Figure 1:**
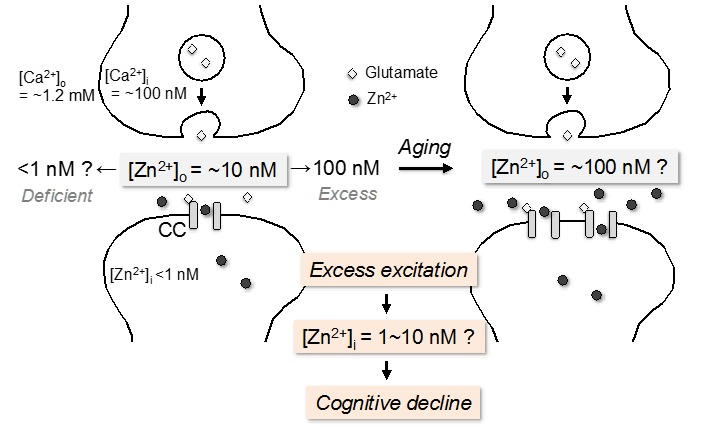
Zn^2+^-mediated cognitive decline via rapid influx of extracellular Zn^2+^. Estimated basal concentration of extracellular Zn^2+^ is ~10 nM in the adult brain. When the basal concentration of extracellular Zn^2+^ reaches 100 nM in the adult brain, it induces cognitive decline. In contrast, even if the basal concentration of extracellular Zn^2+^ reaches 100 nM in the aged brain, it does not induce cognitive decline, suggesting that the basal concentration of extracellular Zn^2+^ is ~100 nM in the aged brain. Extracellular Zn^2+^-mediated cognitive decline is induced by glutamateric synapse excitation, in which intracellular Zn^2+^ may reach 1~10 nM. The synapses are non-zincergic. CC: Ca^2+^-permeable channels.

Divalent cations such as Ca^2+^ and Mg^2+^ are involved in synaptic neurotransmission [24]. Among divalent cations, Ca^2+^ concentration is the highest in brain parenchyma cells and is approximately 1.2 mM in the cerebrospinal fluid (CSF) and brain extracellular fluid in the adult rats (Figure 1) [25]. Approximately 2 mM Ca^2+^ is added to artificial cerebrospinal fluid (ACSF) based on essentiality of intracellular Ca^2+^ signaling in neurons and glial cells [26,27]. However, excess influx of extracellular Ca^2+^ into neurons is linked with the pathophysiological process of neurodegeneration [28,29,30].

Zinc concentration in the CSF is in the range of 150–380 nM [31,32,33]. It is estimated that the basal (static) concentration of extracellular Zn^2+^ is approximately 10 nM in the brain of the adult rats (Figure 1) [34]. A small part of extracellular zinc is free ion (Zn^2+^) in the brain under the basal condition. To research synaptic function, much less attention has been paid to the essentiality of Zn^2+^ in brain extracellular fluid. ACSF, i.e., brain extracellular medium, without Zn^2+^ has been used for in vitro and in vivo experiments. It is likely that not only neuronal excitation but also LTP is modified in brain slices immersed in ACSF without Zn^2+^, in which original neurophysiology might be modified [35,36]. Clarifying the action of extracellular Zn^2+^ in the range of physiological concentrations is important to precisely understand synaptic function. Furthermore, such clarification is also important to understand the bidirectional action of Zn^2+^ under physiological and pathological conditions. It is recognized that low nanomolar concentrations of Zn^2+^ are more physiological than micromolar concentrations of Zn^2+^, which are widely used and often neurotoxic.

Spontaneous presynaptic activity assessed with FM4-64, an indicator of presynaptic activity (exocytosis), in the stratum lucidum where mossy fibers are contained is significantly suppressed in brain slices from young rats immersed in ACSF containing 10 nM Zn^2+^, but not in ACSF containing 10 nM Cu^2+^ or 10 nM Fe^3+^, indicating that hippocampal presynaptic activity is enhanced in brain slices prepared with ACSF without Zn^2+^ [36]. Suh et al. [37] report that acute brain slice preparations are poorly suitable to research the role of endogenous Zn^2+^ released from zincergic neurons. Vesicular Zn^2+^ levels are decreased in the process of slice preparation, and in vitro Zn^2+^ release is reduced to approximately 25% of in vivo Zn^2+^ release. While physiological concentration of extracellular Zn^2+^ is low nanomolar in young rat brain, it may be elevated along with aging (Figure 1), based on the age-related increase in extracellular zinc concentration in the hippocampus [38].

## 3. Impact of Synaptic Zn^2+^ Dynamics on Cognition

Extracellular Ca^2+^ concentration is not affected by neuronal excitation. In contrast, extracellular Zn^2+^ concentration is dynamically increased by zincergic excitation, but not by non-zincergic excitation. In any case, extracellular dynamics of Ca^2+^ and Zn^2+^ is critically linked with their intracellular dynamics. The basal concentration of intracellular (cytosol) Ca^2+^ is 10–100 nM, while that of intracellular Zn^2+^ is extremely low and estimated to be less than 1 nM (Figure 1) [39,40]. While intracellular Ca^2+^ serves as a signaling factor for plastic changes at synapses, intracellular Zn^2+^ is increased for not only signaling for plastic changes during learning and cognitive activity but also plastic changes in synapse structure [41,42]. The optimal range of intracellular Zn^2+^ increased during learning and cognitive activity, which is dynamically linked with Zn^2+^ release at zincergic synapses, remains to be clarified. Even at non-zincergic synapses, postsynaptic intracellular Zn^2+^ may reach ~1 nM and the increase originates in internal stores/proteins unlike the neurotoxic increase via extracellular Zn^2+^ influx as described below.

LTP at zincergic mossy fiber-CA3 pyramidal cell synapses is induced by the presynaptic mechanism, in which glutamate release is persistently increased. Mossy fiber LTP induction critically depends on the rise in presynaptic Ca^2+^ [43,44,45], which activates the calcium-calmodulin-sensitive adenyl cyclase I [46]. Zn^2+^ released from mossy fibers is immediately retaken up into presynaptic terminals through Ca^2+^ channels and activates a Src family kinase, which promotes tropomyosin-related kinase B (TrkB) activation. The activation leads to the phosphorylation and activation of phospholipase Cγ1, followed by calcium-calmodulin-sensitive adenyl cyclase I activation. Zn^2+^ increases presynaptic glutamate release, while it inhibits postsynaptic mechanism of mossy fiber LTP via Zn^2+^ influx [47,48].

LTP at the Schaffer collateral/commissural-CA1 pyramidal cell synapses depends on the postsynaptic activation of NMDA receptors [49]. NMDA receptor activation increases postsynaptic Ca^2+^ concentration, which leads to LTP and LTD. NMDA receptors consist of multiple subclasses [50] and the subtypes have different sensitivities to Zn^2+^, an endogenous blocker [51,52,53,54]. ZnAF-2DA is a useful tool to evaluate the direct involvement of Zn^2+^ in cognitive function. ZnAF-2DA, a membrane-permeable Zn^2+^ indicator, is taken up into neurons through the plasma membrane and is hydrolyzed by esterase in the cytosol, resulting in the production of ZnAF-2, which cannot permeate the plasma membrane [55,56]. When ZnAF-2DA is locally injected into the hippocampal CA1, intracellular ZnAF-2 is detected only in the injected area in the CA1 and can block cellular transients in Zn^2+^ concentration (*K*_d_, 2.7 nM for Zn^2+^). The concurrent evaluations of in vivo LTP and learning behavior in separated experiments using ZnAF-2DA answer whether the in vivo LTP via intracellular Zn^2+^ signaling is linked with learning behavior. The influx of extracellular Zn^2+^ into CA1 pyramidal cells, which is linked with Zn^2+^ release form the zincergic Schaffer collateral, is required for object recognition memory via in vivo Schaffer collateral LTP [57]. Glutamatergic input to CA1 pyramidal cells via the medial perforant pathway (the temporoammonic pathway) from the entorhinal cortex facilitates memory consolidation [58] and is required for temporal association memory [59] and spatial working memory [60]. Although the medial perforant pathway from the entorhinal cortex, is non-zincergic [61], intracellular Zn^2+^ signaling, which originates in internal stores/proteins, is required for LTP at medial perforant pathway-CA1 pyramidal cell synapses [62]. It is likely that intracellular Zn^2+^ signaling in CA1 pyramidal cells is also involved in cognitive function via in vivo perforant pathway LTP.

The lateral and medial entorhinal cortices are connected with the dentate gyrus. The lateral and the medial perforant pathways, which originate in the lateral and the medial entorhinal cortices, respectively, comprise physiologically distinct inputs to the dentate gyrus. The lateral perforant pathway transmits nonspatial information, while the medial perforant pathway transmits spatial information [63]. In regard to LTP at medial perforant pathway-dentate granule cell synapses, calmodulin-dependent protein kinase II *α* (*α*-CaMKII)/brain-derived neurotrophic factor (BDNF) signaling pathway plays a key role for LTP induction. Zinc deficiency-induced cognitive and synaptic impairments are linked with disruption of *α*-CaMKII/BDNF signaling pathway [64]. In dentate granule cells, intracellular Zn^2+^ signaling originates in internal stores/proteins and is necessary for object and space recognition memory via medial perforant pathway LTP [65,66]. In postsynaptic neurons innervated by non-zincergic medial perforant pathway, glutamate receptor activation triggers off Zn^2+^ release from internal stores/proteins that remain to be clarified.

Hippocampal neurogenesis always produces dentate granule cells, in which NMDA receptor-dependent synaptic plasticity is involved in learning and memory [67,68]. Zn^2+^ is concentrated in the dentate gyrus of the hippocampus [69] and is required for neurogenesis process [70]. In human neuronal precursor cells, zinc deficiency induces apoptosis via mitochondrial p53- and caspase-dependent pathways [71], suggesting that dynamic Zn^2+^ transport to neuronal precursor cells is critical for learning and memory via hippocampal neurogenesis [72].

## 4. Impact of Synaptic Zn^2+^ Dynamics on Cognitive Decline

Aging has progressive pathophysiological features and is linked with altered cell metabolism, damaged nucleic acid, oxidative stress, and deposition of abnormal forms of proteins. Aging also is characterized by cognitive decline, neuronal loss, and vulnerability to neurological disorders [73] and may be often related with altered Zn^2+^ homeostasis in the brain [74,75]. Hippocampal zinc concentration is decreased in aging, which decreases zinc transporter-3 (ZnT3) protein. ZnT3 controls synaptic vesicular Zn^2+^ levels. Zn^2+^ release from zincergic neuron terminals, which dynamically modifies the basal concentration of extracellular Zn^2+^, is decreased in aging [74], while the basal concentration of extracellular Zn^2+^ may be increased [38], probably as a compensatory mechanism. A negative modulation of extracellular glutamate signaling by extracellular Zn^2+^ may be involved in cognitive function.

**Figure 2:**
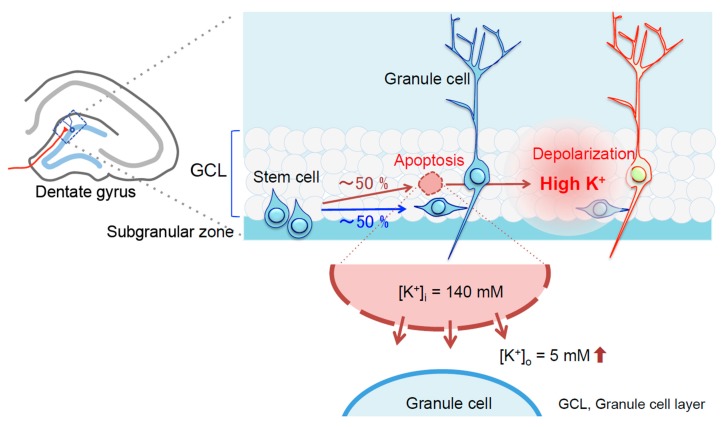
Neuronal depolarization via neurogenesis-related apoptosis. Neurogenesis-related apoptosis increases extracellular K^+^ concentration ([K^+^]_o_), which is due to the efflux of intracellular K^+^ as shown by red arrows, in the dentate granule cell layer and can lead dentate granule cells to depolarization, followed by extracellular Zn^2+^ influx-mediated cognitive decline. The blue arrow shows the process of neurogenesis and red up-arrows show the process of apoptosis and efflux of intracellular K^+^. [K^+^]_i_: intracellular K^+^ concentration.

Metal chaperones i.e., clioquinol and PBT2, prevent normal age-related cognitive decline [76,77], suggest that metal chaperones are effective for preventing Zn^2+^-mediated cognitive decline that is observed in aging and disease. The hippocampus is vulnerable to Zn^2+^ neurotoxicity [78] and the dentate gyrus is the most vulnerable to aging process [2,79]. Although the vulnerability to aging is poorly understood, it is possible that synaptic Zn^2+^ dynamics is involved in the vulnerability. New granule cells are continuously produced in the subgranular zone of the dentate gyrus (Figure 2) and the decreased rate of hippocampal neurogenesis is involved in age-related cognitive decline [80]. Neurogenesis-related apoptosis, which seems to be increased along with aging, always occurs in the dentate gyrus. In the subgranular zone, the apoptosis locally increases extracellular K^+^ and the increase is due to the efflux of intracellular K^+^ (approximately 140 mM) by disruption of the plasma membrane. The increase in extracellular K^+^ may excite granule cells and pyramidal basket cells, which exist nearby in the dentate gyrus, and disturbs intracellular dynamics of Ca^2+^ and Zn^2+^ (Figure 2). As a matter of fact, both memory acquisition via LTP induction and memory retention via LTP maintenance are impaired after local injection of high K^+^ into the dentate gyrus [81,82,83] or the CA1 [84]. The impairments are due to an increase in intracellular Zn^2+^, but not that in intracellular Ca^2+^, because the impairments are rescued with CaEDTA, which forms membrane-impermeable ZnEDTA in the extracellular compartment and inhibits the influx of extracellular Zn^2+^, but not that in extracellular Ca^2+^ [82]. The evidence indicates Zn^2+^-mediated cognitive decline via transient Zn^2+^ accumulation in dentate granule cells (Figure 1) and CA1 pyramidal cells.

If the basal level of extracellular Zn^2+^ is increased age-dependently in the hippocampus (Figure 1) [38], it is estimated that Zn^2+^-mediated cognitive decline more readily occurs in the aged brain. High K^+^-induced increase in intracellular Zn^2+^ is facilitated in the aged dentate gyrus and leads to attenuating both LTP induction and maintained LTP at medial perforant pathway-dentate granule cell synapses of aged rats [38,83], suggesting that the influx of extracellular Zn^2+^ into dentate granule cells more readily occurs in aged rats and is a cause of age-related cognitive decline via attenuation of LTP. It is likely that neurogenesis-related apoptosis is involved in Zn^2+^-mediated cognitive decline.

GluR2-lacking calcium-permeable *α*-amino-3-hydroxy-5-methyl-4-isoxazolepropionate (AMPA) receptors are involved in Zn^2+^-mediated neurodegeneration in the hippocampal CA1 and CA3 [18,85,86]. In the hippocampus, the levels of GluR1 and GluR2 mRNA are highest in the dentate gyrus and the GluR1/GluR2 mRNA ratios are elevated along with aging [87]. The findings suggest that Zn^2+^ influx through Ca^2+^-permeable AMPA receptors, which more readily occurs in the aged dentate gyrus, plays a key role for cognitive decline [38,83]. Intracellular Zn^2+^ can reach approximately 10 nM via the rapid influx of extracellular Zn^2+^ (Figure 1) [34]. Both increases in extracellular Zn^2+^ and Ca^2+^-permeable AMPA receptors contribute to Zn^2+^-mediated cognitive decline in aging.

**Figure 3:**
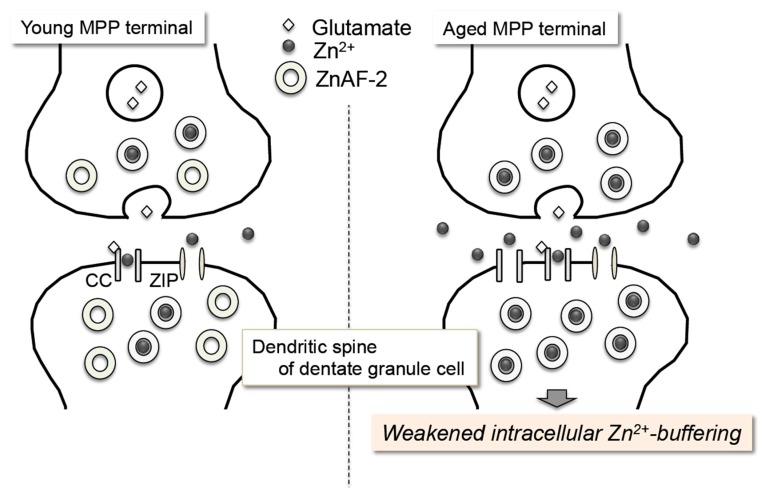
Is intracellular Zn^2+^-buffering weakened in the dentate gyrus along with aging? In vivo intracellular Zn^2+^-buffering is assessed in the dentate molecular layer where non-zincergic media performant pathway (MPP)-dentate granule cell synapses are contained. Intracellular ZnAF-2, an index of intracellular Zn^2+^-buffering capacity, can bind Zn^2+^ at young MPP synapse 2 h after ZnAF-2DA injection into the dentate molecular layer, but not at aged MPP synapses. Capacity of intracellular ZnAF-2 for binding intracellular Zn^2+^ is more rapidly lost in in aged dentate gyrus, probably due to easiness of extracellular Zn^2+^ influx, suggesting a reduced capacity of intracellular Zn^2+^-buffering in aged dentate gyrus. ZIP: Zrt/Irt-like proteins.

Although intracellular Zn^2+^ level in the process of LTP maintenance is unknown, LTP maintenance at medial perforant pathway-dentate granule cell synapses is affected by chelation of intracellular Zn^2+^ with intracellular ZnAF-2 [66] and the aged dentate gyrus is more susceptible to the chelating effect on LTP maintenance [83]. When ZnAF-2DA is used as an index of the capacity binding intracellular Zn^2+^, interestingly, the capacity of intracellular ZnAF-2 for binding intracellular Zn^2+^ is more rapidly lost in the aged dentate molecular layer where medial perforant pathway-dentate granule cell synapses are contained than in the young dentate molecular layer, suggesting that intracellular Zn^2+^-buffering is weakened in the dentate gyrus along with aging (Figure 3) [83]. Characteristics (easiness) of extracellular Zn^2+^ influx may be linked with weakened intracellular Zn^2+^-buffering in the aged dentate gyrus [28]. Although the actual state of intracellular Zn^2+^-buffering is poorly understood, Ca^2+^-permeable channels, zinc transporters (ZIP and ZnT), zinc-binding proteins such as metallothioneins, and Zn^2+^-containing internal stores are involved in the Zn^2+^-buffering system.

## 5. Perspectives

Vulnerability to Ca^2+^ dysregulation has been observed in the process of brain aging [88,89,90]. It has been reported that Ca^2+^ dysregulation is not ubiquitous. The mechanisms of dysregulation are observed in specific cell populations and areas in the brain. For example, L-type Ca^2+^ channels is age-dependently increased in hippocampal pyramidal cells [91]. Age-dependent reduction in the NMDA receptor function is observed in the hippocampus and the frontal cortex [92], suggesting a compensatory mechanism to availability/restriction for intracellular Ca^2+^ signaling. Intracellular Ca^2+^-buffering, which is involved in cognitive function, is weakened during brain aging [89]. In contrast, intracellular Zn^2+^-buffering is also dynamically involved in cognition and its decline. However, the Zn^2+^-buffering system is more poorly understood than the Ca^2+^-buffering system, and its clarification is required for understanding cognition and its decline.
